# Carbapenemase-producing *Pseudomonas aeruginosa* –an emerging challenge

**DOI:** 10.1080/22221751.2022.2048972

**Published:** 2022-03-12

**Authors:** Fred C. Tenover, David P. Nicolau, Christian M. Gill

**Affiliations:** a Cepheid, Sunnyvale, CA, USA; b Center for Anti-Infective Research and Development, Hartford Hospital, Hartford, CT, USA; c Division of Infectious Diseases, Hartford Hospital, Hartford, CT, USA

**Keywords:** *Pseudomonas aeruginosa*, carbapenems, carbapenemase, susceptibility testing, beta-lactamase, beta-lactamase inhibitor

## Abstract

Carbapenem-resistant *Pseudomonas aeruginosa* (CR-PA) is a major healthcare-associated pathogen worldwide. In the United States, 10–30% of *P. aeruginosa* isolates are carbapenem-resistant, while globally the percentage varies considerably. A subset of carbapenem-resistant *P. aeruginosa* isolates harbour carbapenemases, although due in part to limited screening for these enzymes in clinical laboratories, the actual percentage is unknown. Carbapenemase-mediated carbapenem resistance in *P. aeruginosa* is a significant concern as it greatly limits the choice of anti-infective strategies, although detecting carbapenemase-producing *P. aeruginosa* in the clinical laboratory can be challenging. Such organisms also have been associated with nosocomial spread requiring infection prevention interventions. The carbapenemases present in *P. aeruginosa* vary widely by region but include the Class A beta-lactamases, KPC and GES; metallo-beta-lactamases IMP, NDM, SPM, and VIM; and the Class D, OXA-48 enzymes. Rapid confirmation and differentiation among the various classes of carbapenemases is key to the initiation of early effective therapy. This may be accomplished using either molecular genotypic methods or phenotypic methods, although both have their limitations. Prompt evidence that rules out carbapenemases guides clinicians to more optimal therapeutic selections based on local phenotypic profiling of non-carbapenemase-producing, carbapenem-resistant *P. aeruginosa*. This article will review the testing strategies available for optimizing therapy of *P. aeruginosa* infections.

## Introduction

Carbapenem-resistant *Pseudomonas aeruginosa* (CR-PA) is a major healthcare-associated pathogen worldwide [1–3]. In the United States, *P. aeruginosa* is the primary cause of ventilator-associated pneumonia (VAP) in long-term acute care hospitals and on hospital wards and second most common cause of VAP in intensive care units. It also is the third most common cause of catheter-related urinary tract infections [4]. Overall, in the United States, 10–30% of *P. aeruginosa* isolates are carbapenem-resistant [5,6] while globally the percentage varies considerably. There are several key mechanisms of carbapenem resistance in *P. aeruginosa*. The first mechanism is efflux of the drug, which is mediated by overexpression of the *MexAB-OprM* efflux pump [7]. This results in resistance to most beta-lactam drugs with the exception of imipenem. The second mechanism is the overproduction of AmpC beta-lactamase and inactivation of the *OprD* outer membrane protein. This combination of mechanisms can cause resistance to essentially all antipseudomonal beta-lactams. A less common mechanism of carbapenem resistance among *P. aeruginosa* isolates, but one that appears to be increasing in frequency, is the production of carbapenemases [2,8,9]. This mechanism of carbapenem resistance is important because it significantly alters the efficacy of commonly used antipseudomonal agents, including ceftazidime, cefepime, piperacillin-tazobactam, as well as the newly introduced beta-lactam/beta-lactamase inhibitor combinations such as ceftolozane-tazobactam, imipenem-relebactam and ceftazidime-avibactam. The carbapenem resistance determinants carried by *P. aeruginosa* are often encoded on plasmids, such as IncP type; class I integrons, such as those carrying the *bla*_VIM_ gene; and other mobile genetics elements, such as those associated with insertion sequences with a common region (ISCRs), which enhance the organism's ability to disseminate resistance among multiple species [10]. In addition, these isolates frequently carry additional resistance determinants that diminish the clinical utility of the fluoroquinolones and aminoglycosides. Carbapenemase-producing *P. aeruginosa* (CP-PA) are often resistant to all of these therapeutic options, thus making treatment failure a likely outcome. CP-PA has also been associated with nosocomial spread prompting infection prevention interventions [11].

## Epidemiology of carbapenemase-producing *P. aeruginosa*

*P. aeruginosa* isolates have been reported to contain a wide variety of carbapenemases globally. For example, in Latin America, this includes KPC, GES, IMP, VIM, NDM, and SPM [9]. In the Arabian Peninsula, carbapenemases in *P. aeruginosa* include VIM, IMP, and NDM [8]. In the United States, carbapenemases in *P. aeruginosa* include KPC, NDM, VIM, and IMP [12,13]. Unfortunately, several of the phenotypic methods for detecting carbapenemases that have been used worldwide, such as the Modified Hodge test, show either poor sensitivity or specificity, which confounds the epidemiology of these organisms [14]. This and the lack of testing specifically for carbapenemase production among CR-PA globally suggest that the prevalence of CP-PA may be much higher than is perceived. The diversity and emerging prevalence of carbapenemase producers among CR-PA has been recently highlighted in the multi-national ERACE-PA Surveillance Program [14]. Of the 807 CR-PA collected over 2019–2021 from 17 centres in 12 countries, 33% tested carbapenemase-positive phenotypically (using the mCIM method) and of these, 86% were genotypically positive with the most common being VIM followed by GES. While carbapenemase producers were anticipated in the Middle East centres based on previously published epidemiology data, a high prevalence and diversity was also observed in the European, South American, and African centres. Moreover, in the United States centres (*n* = 5), a region not known to be of high prevalence, CP-PA were identified in 3–30% of the CR-PA. These contemporary global data suggest that carbapenemase testing in CR-PA is warranted. The key question is whether laboratories should be testing either for carbapenemase production phenotypically or genotypically via PCR for the presence of specific carbapenem-resistance genes among CR-PA isolates to assist antimicrobial stewardship programmes in selecting appropriate therapy for pseudomonal infections.

## Testing carbapenemase-producing *P. aeruginosa* to aid antimicrobial stewardship

At present, when a CR-PA isolate is identified in a clinical laboratory during the first round of antimicrobial susceptibility testing, many institutions will perform additional susceptibility tests for ceftolozane/tazobactam, ceftazidime/avibactam, and/or imipenem/relebactam using automated antimicrobial susceptibility testing (AST) methods, agar diffusion methods (i.e. Gradient diffusion strips), or disk diffusion to guide therapy. These antimicrobial agents are highly active against a wide variety, although not all, CR-PA [15–17]. However, not all laboratories have access to susceptibility testing panels, strips or disks for these beta-lactam/beta-lactamase inhibitor combinations. In addition, gradient diffusion strips and disk diffusion testing for these novel antimicrobials require another 16–20 h of incubation after the initial susceptibility test results become available, which may slow the decision-making process for guiding therapy. More timely data, such as that provided by commercial PCR or immunochromatographic tests to exclude the most common carbapenemases, can guide clinicians to early use of ceftolozane/tazobactam, ceftazidime/avibactam, and imipenem/relebactam as they are highly active against non-carbapenemase-producing, CR-PA. On the other hand, detection of carbapenemases, especially metallo-beta-lactamases (e.g. IMP, NDM, and VIM) will indicate the need to consider cefiderocol [1,18] or combination therapy inclusive of aztreonam [19–21].

**Figure 1.**
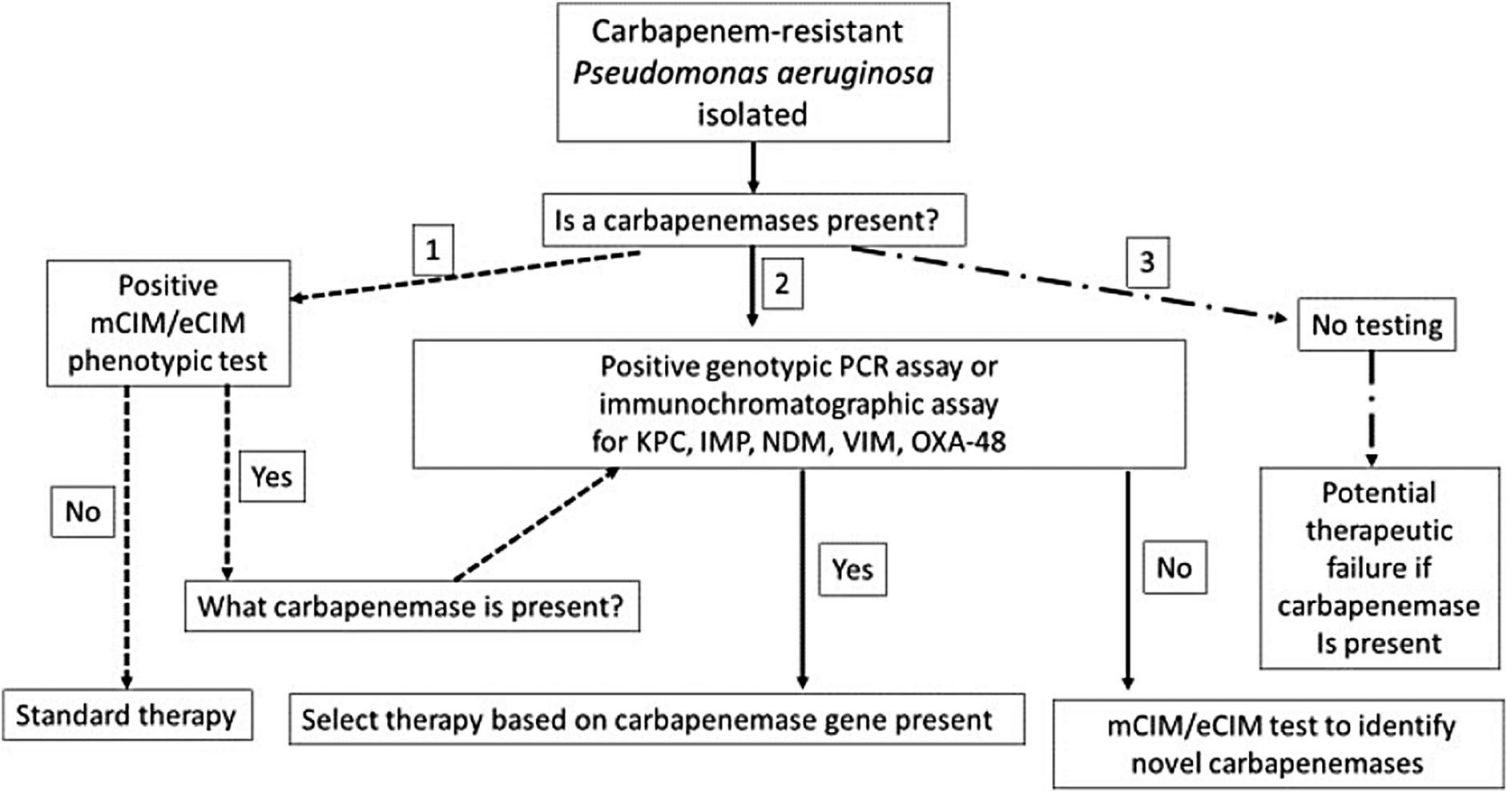
Laboratory-based screening options for the detection of carbapenemase-producers among carbapenem-resistant *P. aeruginosa*.

How should laboratories test for CP-PA? There are two approaches to testing that could guide antimicrobial stewardship efforts and thus improve therapeutic outcomes (Figure 1, options 1 and 2). The first approach (Option 1) is to test colonies of *P. aeruginosa* with a broad phenotypic carbapenemase test, such as the modified carbapenem inactivated method (mCIM) or CarbaNP test [22,23]. These tests indicate whether or not a carbapenemase is present in the isolate, but not which type of carbapenemases it is (i.e. a serine versus metallo-beta-lactamase). However, performing a second mCIM test in the presence of EDTA (i.e. the eCIM test) can differentiate serine carbapenemases (which are not inhibited by EDTA) from metallo-carbapenemases, which is key information for selecting anti-pseudomonal therapy. Alternatively, if only the mCIM test is performed and it is positive, a genotypic test, either PCR or an immunochromatographic test, can be used to identify the specific classes of carbapenemases present (i.e. KPC, VIM, IMP, VIM, and OXA-48) [24–26]. Several commercial tests have been shown to be accurate for detection of carbapenemases or carbapenem resistance genes, although the costs of the tests may vary from country to country. It should be noted that the mCIM test can have difficulty detecting some carbapenemases, such as IMP [27,28], although a recent study showed the combination of mCIM and eCIM testing to be effective for detecting most other carbapenemases in *P. aeruginosa* isolates [29].

The second testing option is to begin with a commercial PCR or immunochromatographic test that detects KPC, VIM, IMP, VIM, and OXA-48, and if that test is negative to follow up with the mCIM or mCIM/eCIM combination tests. The advantage of the latter approach is that both the PCR and immunochromatographic test can often be completed in under 1 h, which although potentially more expensive for the laboratory, can lead to more precise therapeutic interventions in a time frame that may be 48 h sooner than Option 1. This makes the up-front use of these methods cost-effective. While whole genome sequencing (WGS) of isolated colonies can provide much greater information about the mechanisms of antimicrobial resistance in a *P. aeruginosa* isolate compared with the phenotypic and genotypic methods mentioned above, the slow turn-around time of results, the technical expertise required for nucleic acid extraction, library preparation, sequencing, and finally the lack of standardized databases to translate genotypes into phenotypes that can be readily understand by clinicians, currently limit the use of WGS to research facilities rather than hospital laboratories.

Option 3 is to perform to no additional testing and treat empirically, but this is not recommended as the number of treatments failures will surely increase as CP-PA isolates continue to spread globally. While the argument to forgo additional phenotypic and genotypic assessments for carbapenemase-production in CR-PA often focuses on the perceived value of the test relative to percent positive, staff and testing resources, the clinical implementation of a recently developed algorithm will aid in streamlining carbapenemase detection workflow in the laboratory [27]. Importantly, test availability, whether positive or negative, translates to actionable results in the form of enhanced therapeutic and / or infection control interventions that are central to safeguarding good clinical outcomes while minimizing the dissemination of CP-PA.

## Summary

There is an increasing probability of treatment failures with infections caused by carbapenem-resistant *P. aeruginosa* due to the unrecognized presence of carbapenemases. Combining both phenotypic and genotypic methods can significantly shorten the time to effective therapy and enhance patient outcomes. Yet, these tests often are not performed on *P. aeruginosa* isolates in many clinical laboratories. Thus, optimizing therapy for *P. aeruginosa* infections remains a challenge.
